# Genetic analysis of osteopetrosis in Pakistani families identifies novel and known sequence variants

**DOI:** 10.1186/s12920-021-01117-4

**Published:** 2021-11-09

**Authors:** Chunyu Liu, Muhammad Ajmal, Zaineb Akram, Tariq Ghafoor, Muhammad Farhan, Sobia Shafique, Sughra Wahid, Shahar Bano, Jianqiu Xiao, Humayoon Shafique Satti, Feng Zhang, Tahir Naeem Khan

**Affiliations:** 1 Obstetrics and Gynecology Hospital, NHC Key Laboratory of Reproduction Regulation (Shanghai Institute for Biomedical and Pharmaceutical Technologies), School of Life Sciences, Fudan University, Shanghai, 200011 China; 2 Institute of Biomedical and Genetic Engineering, Islamabad, 44000 Pakistan; 3 Armed Forces Bone Marrow Transplant Centre, CMH Medical Complex, Rawalpindi, 46000 Pakistan; 4 KRL General Hospital, Islamabad, 44000 Pakistan; 5 Division of Bone and Mineral Diseases, Musculoskeletal Research Center, Washington University School of Medicine, St. Louis, MO 63110 USA; 6 Department of Biological Sciences, National University of Medical Sciences, Rawalpindi, 46000 Pakistan; 7 National Institute of Advance Studies and Research, National University of Medical Sciences, Rawalpindi, 46000 Pakistan

**Keywords:** Autosomal recessive osteopetrosis, Genetic diagnosis, TCIRG1, Disease variants

## Abstract

Osteopetrosis is a genetically heterogenous, fatal bone disorder characterized by increased bone density. Globally, various genetic causes are reported for osteopetrosis with all forms of inheritance patterns. A precise molecular diagnosis is necessary for prognosis and for prescribing treatment paradigms in osteopetrosis. Here we report on thirteen individuals diagnosed with infantile malignant osteopetrosis coming from ten unrelated Pakistani families; nine of whom are consanguineous. We performed whole exome sequencing and Sanger sequencing in all families and identified homozygous variants in genes previously reported for autosomal recessive inheritance of osteopetrosis. All the identified variants are expected to affect the stability or length of gene products except one nonsynonymous missense variant. *TCIRG1* was found as a candidate causal gene in majority of the families. We report six novel variants; four in *TCIRG1* and one each in *CLCN7* and *OSTM1*. Our combined findings will be helpful in molecular diagnosis and genetic counselling of patients with osteopetrosis particularly in populations with high consanguinity.

**Supplementary Information:**

The online version contains supplementary material available at 10.1186/s12920-021-01117-4.

## Introduction

Osteopetrosis is a group of clinically and genetically heterogeneous disorders of increased bone density caused by defective osteoclast function and decreased bone resorption, making bones fragile and prone to fractures [1]. Several types of osteopetrosis have been described in literature, mainly distinguished on the basis of pattern of inheritance; autosomal dominant, autosomal recessive and X-linked or intermediate autosomal type [2]. Autosomal recessive osteopetrosis (ARO), also known as infantile malignant osteopetrosis is a rare phenotype with an estimated incidence of 1 in 250,000 live births [3] although as in the case with other autosomal recessive genetic disorders, the incidence of ARO is expected to be higher in highly consanguineous populations. The symptoms of ARO start appearing soon after birth and often proves to be fatal if not treated early. Symptoms arising from a reduced marrow compartment include anemia, thrombocytopenia and infections along with hepatosplenomegaly. An increased density of skull bone results in neurodegeneration and compression of optic nerve resulting in blindness and deafness [4].

ARO is caused by biallelic mutations in one of the at least seven genes namely *TCIRG1, CLCN7, OSTM1, SNX10, PLEKHM1, TNFSF11, TNFRSF11A* [5]. Mutations in genes *TCIRG1, CLCN7, OSTM1, SNX10 and PLEKHM1* lead to osteoclast rich osteopetrosis, which has abundant but nonfunctional osteoclasts [6]. These genes encode proteins that are involved in acidification of the resorption lacunae and/or vascular transport. Mutations in *TNFSF11* and *TNFRSF11A* result in blockage of osteoclastogenesis which is osteoclast poor ARO [7].

Approximately half of the ARO cases are caused by mutations in *TCIRG1* followed by *CLCN7* mutations which are associated with 17.5% of ARO cases [8]. More than hundred candidate disease causal variants have been reported in *TCIRG1* (The Human Gene Mutation Database), majority in Western populations. The mutational spectrum of ARO is underreported in a highly consanguineous Pakistani population. In current study, we report genetic analysis of ARO in ten unrelated families originating from Pakistan.

## Methods

### Human research participants

This study includes total of 13 affected individuals from ten unrelated families afflicted with osteopetrosis. Nine of these families are consanguineous while for one family the consanguinity is not known. Study participants originate from different regions of Pakistan. Studies and procedures related to clinical examination, human sample collection and genetic analysis were approved by Institutional Review Boards and Ethical committees of the Armed Forces Bone Marrow Transplant Centre, Rawalpindi, Pakistan (families OP1, OP2, OP3, OP4) and Institute of Biomedical and Genetic Engineering, Islamabad, Pakistan (families OP5, OP6, OP7, OP8, OP9, OP10). Signed informed consent was obtained from all participants or their legal representatives for study procedures and publication of clinical and genetic findings. Subsequent to informed consent, we obtained peripheral blood samples from affected individuals and their healthy family members by standard venipuncture. We extracted DNA using a phenol–chloroform extraction method or QIAamp DNA Maxi Kit (Qiagen).

### Genetic analysis

We performed whole exome sequencing (WES) in eight families, on genomic DNA obtained from an affected individual from each family except family OP4 where we performed WES in both affected individuals in the family. Genomic DNA was target enriched by the Agilent SureSelect^XT^ Human All Exon Kit. Next-generation sequencing was conducted with the Illumina HiSeq X-TEN platform at Cloud Health Genomics. Reads were aligned to the human genome reference assembly (UCSC Genome Browser hg19) with BWA. Picard software was employed to remove PCR duplicates and evaluate the quality of variants to attaining effective reads, quality bases, average coverage depth and coverage ratio. Single-nucleotide variants (SNVs) and indels were called and analyzed with GATK using an in-house variant filtration pipeline. We then used ANNOVAR for functional annotation with OMIM, Gene Ontology, KEGG Pathway, SIFT, PolyPhen-2 and MutationTaster.

In WES-analyzed families, variants generated by WES were assessed and filtered for rare variants with a minor allele frequency (MAF) of < 0.01 in relevant ethnicity-matched populations in control databases (including the Genome Aggregation Database; gnomAD, the Exome Aggregation Consortium; ExAC, and the 1000 Genomes Project). Non-synonymous, exonic or splice variants, conforming to recessive pattern of inheritance of disease in each family were prioritized as candidates. We performed bidirectional Sanger sequencing with BigDye terminator v3.1 cycle sequencing chemistry to confirm candidate variants and segregation in all available family members.

In two additional families (OP5 and OP9), we performed bidirectional Sanger sequencing of previously published genes associated with autosomal recessive osteopetrosis starting from sequencing of the most frequently associated genes such as *TCIRG1* and *CLCN7*. For this purpose, primers were designed covering all the exons and exon–intron boundaries of candidate genes. PCR amplified, purified samples were run on ABI 3500 Genetic Analyzer (Applied Biosystems) and sequence chromatogram was analyzed using Sequencing Analysis Software v6.0 (Applied Biosystems).

## Results

### Clinical features of the osteopetrosis patients (Table 1)

**Table 1:** Clinical features of individuals with osteopetrosis

	OP1-2	OP2-4	OP2-5	OP3-3	OP4-3	OP4-4	OP5-2	OP6-3	OP6-5	OP7-3	OP8-3	OP9-3	OP10-3
Gender	M	M	M	F	M	M	F	M	F	F	M	CVS	M
Age at diagnosis (months)	09	01	01	01	03	01	At birth	2.5	01	03	1.5	-	07
Parents are related	Yes	Yes	Yes	Yes	Yes	Yes	Yes	No	No	Yes	Yes	Yes	Yes
Another affected sibling	No	Yes	Yes	Yes	Yes	Yes	Yes	Yes	Yes	No	No	Yes	No
Delayed developmental milestones	Yes	No	Yes	Yes	Yes	Yes	Yes	No	No	No	Yes	–	No
Macrocephaly	No	No	No	Yes	Yes	Yes	Yes	No	No	No	Yes	–	
Hepatosplenomegaly	Yes	Yes	Yes	No	Yes	Yes	Yes	Yes	Yes	Yes	Yes	–	Yes
Impairments visual, hearing, speech	Hearing	Visual	Visual	Visual	Visual	No	Visual and hearing	No	No	No	No	–	Visual and hearing
Anemia	Yes	Yes	Yes	Mild	Yes	Yes	Yes	Yes	Mild	Yes	No	–	Severe
Thrombocytopenia	Yes	Yes	Yes	Yes	Yes	Yes	Yes	No	No	Yes	Yes	–	Yes
Required transfusions	Yes	Yes	No	No	No	Yes	No	Yes	No	Yes	No	–	Yes

*M* male, *F* female, *CVS* chorionic villus sample, – not available

#### OP1

The affected child (OP1-2), deceased now, was born to consanguineous parents. He was the youngest of the three siblings. He was deaf and mute since birth and was diagnosed with osteopetrosis at the age of nine months, when he presented with progressive pallor, bicytopenia, hepatosplenomegaly, delayed developmental milestones and failure to thrive. Skeletal survey revealed generalized increase in bone density suggestive of marble bone disease. Due to severe anemia and thrombocytopenia, allogeneic stem cell transplantation was planned after HLA-matching with elder sister (OP1-1). However, the patient succumbed to infections and died before hematopoietic stem cell transplantation (HSCT).

#### OP2

The parents in OP2 family are first-cousins. Their two male children (OP2-4 and OP2-5) are affected with infantile malignant osteopetrosis, whereas the eldest female sibling (OP2-3) is healthy. The elder of the two siblings (OP2-4) presented with severe anemia and bruises in the first month after birth. Bone marrow aspirate from OP2-4 revealed hypocellularity with depressed erythropoiesis. Skeletal survey showed increased bone density and was provisionally labelled as marble bone disease. Due to severe anemia and thrombocytopenia, he was receiving regular blood transfusion since then. He underwent splenectomy at the age of 5 years. He got HLA matched with the father. However, he died at the age of six and a half years before HSCT due to transfusion-associated sepsis.

Considering family history, when the youngest sibling (OP2-5) was born, his chest X-ray and X-ray of long bones were performed at age one month, which showed increased bone density and reduced medullary cavity, suggestive of osteopetrosis. His growth milestones are delayed for his age. He has poor vision, but has some perception of light, whereas hearing is intact. Though his blood parameters are on the lower side but he never received any transfusion. He got HLA-matched with the sister and underwent HSCT at the age of nine months. He is currently one year post-HSCT and off medication.

#### OP3

The parents in this family are first-cousins and they have two children affected with osteopetrosis. One of the affected child died at the age of three years due to complications of osteopetrosis. The details of clinical history are not available for the deceased child. In other affected child (OP3-3), skeletal scan confirmed marble bone disease. She has enlarged skull and delayed growth milestones. She suffers from poor dentition and have difficulty in chewing food. Her vision is markedly impaired, though she is able to follow light. Her blood counts are stable at slightly below normal limits and she has no blood transfusion history.

#### OP4

This consanguineously married couple has two children (OP4-3 and OP4-4) affected with osteopetrosis, currently aged four and two and a half years, respectively. Both children have delayed growth landmarks, frontal bossing of skull, proptosis, hepatosplenomegaly and bicytopenia. The elder sibling has bilateral blindness with intact hearing. Bone scan in both children revealed marble bone disease.

#### OP5

Family OP5 had two affected children, both of them deceased now. One male child died at an early age having severe symptoms of osteopetrosis. At the time of birth, he had macrocephaly, hepatosplenomegaly, blindness with exophthalmoses and deafness. He also presented with severe anemia due to bone marrow failure along with other respiratory problems, which possibly led to his demise. Genetic screening was not performed in this patient.

The second patient (OP5-2) in this family was a female baby who survived till the age of one year. She manifested feeding problems since her birth. Other clinical features of the osteopetrosis were also obvious from the time of birth. These included prominent macrocephaly, skeletal deformities including thick and dense skull bones, neurological abnormalities, frontal bossing and hypertelorism, nystagmus with complete visual loss, deafness, hepatosplenomegaly, bone marrow suppression resulting in severe cytopenia. The serum calcium level was markedly low. She eventually died due to hematological complications.

#### OP6

The family has two affected children born to non-consanguineous couple. Individual OP6-5, deceased now, initially presented with mild splenomegaly. Computed tomography scan of orbit and brain showed increased bone density in OP6-5 which was further confirmed by X-ray scan showing generalized increase in bone density of all bones. Other clinical features include bone within bone appearance and vertebra within vertebra appearance in spine and dense skull bones with sclerotic base. His sample was collected for genetic screening, before he died at the age of 5 years.

Clinical assessment of second affected child (OP6-3) in this family reported occipitofrontal circumference of 43 cm, proptosis, pallor, and no evidence of cranial nerve involvement. Massive hepatosplenomegaly was also observed; dentition was found to be normal for age. Her X-ray scans confirmed increased bone density; indicative of osteopetrosis.

#### OP7

The parents in this family are first cousins. Their only child (OP7-3) deceased now, was diagnosed with osteopetrosis at the age of three months. She presented with pallor, bicytopenia and hepatosplenomegaly. She was initially screened for beta-thalassemia and was found heterozygous for Cd 15 (G > A) mutation in *HBB* inherited from her healthy mother. Bone marrow biopsy revealed 2% blast cells and fragmented red blood cells. Radiological evidence was inconclusive for establishing diagnosis of osteopetrosis, therefore whole exome sequencing was performed in affected child to help diagnose the disease at molecular level.

#### OP8

The proband (OP8-3) in this family, deceased now, was born to consanguineous parents. Hematological investigations of OP8-3 showed lymphocytes with atypical morphology and nucleated red blood cells, decreased platelet count and increased reticulocyte count. This was accompanied by moderate hepatosplenomegaly. Radiological scan revealed increase bone density with metaphyseal lucent bands and increased skull size for age indicative of marble bone disease. No visual and hearing impairments were observed at the time of diagnosis.

#### OP9

The parents in this family are related to each other. Previously they had two children (both deceased) affected with osteopetrosis, but we could not gather detailed clinical history for them. The family came to us with a chorionic villus sample (CVS) from fetus for prenatal genetic screening of osteopetrosis. No clinical details were possible for OP9-3, as it was a fetal sample. We screened all three DNA samples (both parents and fetal sample) for variation in osteopetrosis candidate genes through bidirectional Sanger sequencing to identify the disease causing mutation in the family. Sanger sequencing identified a homozygous mutation (c.2416T > A) in *CLCN7* in the DNA from CVS while both parents were found heterozygous for the said mutation. The fetus was later aborted due to findings consistent with osteopetrosis.

#### OP10

The proband (OP10-3) born to consanguineous parents is a seven month old baby boy, a diagnosed patient of osteopetrosis with congenital heart disease and atrial septal defect. X-ray scan of proband showed bone-in-bone appearance with prominent dense bones and frontal bossing. Other findings included hepatosplenomegaly, progressive vision and hearing loss and bilateral nystagmus. Patient has history of anemia and blood transfusion.

### Genomic studies identify recessive variants in known osteopetrosis genes

To identify the genetic cause(s) of osteopetrosis, we performed WES in one affected individual from families OP1, OP2, OP3, OP6, OP7, OP8, OP10 and two affected individuals in family OP4. We obtained a mean target coverage of 124.61–155.93 with a mean of 93.2% of bases covered > 20X for all affected individuals (Additional file 1: Table 1).

We filtered rare variants that were homozygous and compound heterozygous in affected individuals using a MAF < 1%. None of the compound heterozygous changes fulfilled our filtration criteria. However, we identified homozygous changes in each family in at least one gene known to cause osteopetrosis in multiple studies. Additionally, disease candidate gene sequencing via Sanger sequencing in further two families (OP5 and OP9) with osteopetrosis identified two homozygous variants, one each in *TCIRG1* and *CLCN7.*

**Fig. 1:**
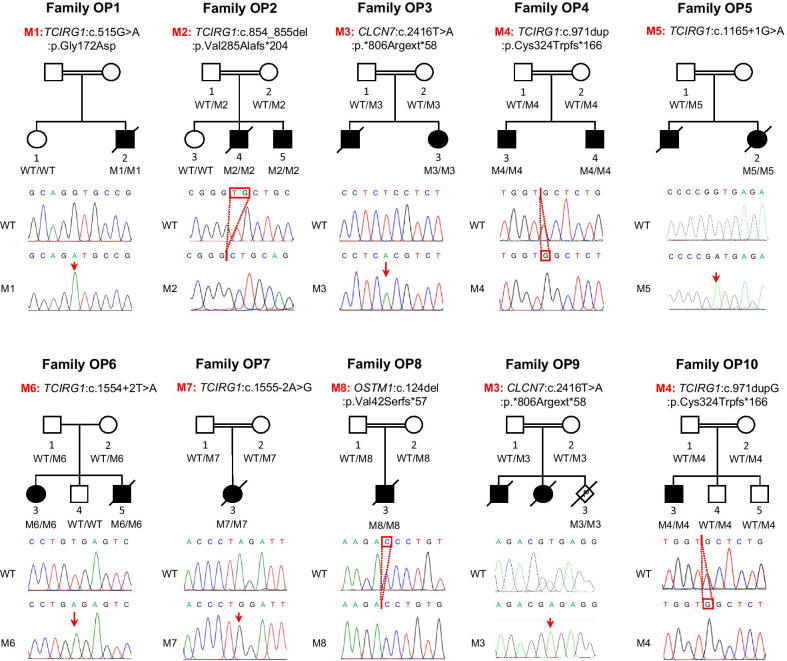
Pedigrees and genetic findings in families with osteopetrosis. Pedigrees of ten Pakistani families (OP1–OP10) with osteopetrosis that segregate as an autosomal recessive trait. Top bold text represent the name of each family and their corresponding sequence variants in osteopetrosis genes shown below. Filled circles or squares indicate affected females and males, respectively. Double lines indicate consanguinity. Individuals with DNA available are indicated with digits and their genotype for corresponding sequence variant is shown below. WT, Wild type; M1–M8, mutation 1- mutation 8. Representative chromatograms showing the pedigree specific sequence variant from a healthy/control wild type (top) and affected homozygous individual (bottom). Position of the sequence variant is indicated with red arrow or red rectangle

In total, seven families were identified with six different homozygous variants in *TCIRG1*, two families with the same homozygous variant in *CLCN7* and a family with homozygous variant in *OSTM1* (Fig. 1). Parents of the affected cases were asymptomatic and where available for genetic analysis, were found to be heterozygous for identified variants. Based on the relevance of identified gene variants to phenotype, segregation with disease, population frequencies and absence of homozygous changes in the general population, we considered these variants as likely cause of osteopetrosis in our families. Variants identified in this study were further characterized based on the variant interpretation guidelines of The American College of Medical Genetics and Genomics [9] (Table 2).

**Table 2:** List of potential disease causal variants identified in this study

Family ID	Genomic change (GRCh37/hg19)	Canonical transcript change	Function	MAF gnomAD^†^ SAS	MAF gnomAD Global	Evidence of pathogenicity^§^	Variant classification^§^	Reference
OP1	Chr11:67810849G > A	*TCIRG1*:NM_006019.4:c.515G > A:p.Gly172Asp	Nonsynonymous SNV	–	–	PM2 PP3 PP4	Uncertain significance	This study
OP2	Chr11:67811644-67811645delGT	*TCIRG1*:NM_006019.4:c.854_855del:p.Val285Alafs*204	Frameshift deletion	–	–	PVS1 PM2 PM4 PP1 PP3 PP4	Pathogenic	This study
OP3	Chr16:1496634A > T	*CLCN7*:NM_001287.6:c.2416 T > A:p.*806Argext*58	Stoploss	–	–	PM2 PM4 PP3 PP4	Likely pathogenic	This study
OP4	Chr11:67811761insG	*TCIRG1*:NM_006019.4:c.971dup:p.Cys324Trpfs*166	Frameshift insertion	–	–	PVS1 PM2 PS1 PP1 PP3 PP4	Pathogenic	Sobacchi et al. [11]
OP5	Chr11:67812570G > A	*TCIRG1*:NM_006019.4:c.1165 + 1G > A	Splicing	–	–^¶^	PVS1 PM2 PP3 PP4	Pathogenic	This study^¶¶^
OP6	Chr11:67815441T > A	*TCIRG1*:NM_006019.4:c.1554 + 2T > A	Splicing	0.0002	0.00002	PVS1 PM2 PP1 PP3 PP4	Pathogenic	Sobacchi et al. 11
OP7	Chr11:67816344A > G	*TCIRG1*:NM_006019.4:c.1555-2A > G	Splicing	–	–^¶¶¶^	PVS1 PM2 PP3 PP4 PP5	Pathogenic	This study^¶¶¶¶^
OP8	Chr6:108395732delC	*OSTM1*:NM_014028.4:c.124del:p.Val42Serfs*57	Frameshift deletion	–	–	PVS1 PM2 PP3 PP4	Pathogenic	This study
OP9	Chr16:1496634A > T	*CLCN7*:NM_001287.6:c.2416T > A:p.*806Argext*58	Stoploss	–	–	PM2 PM4 PP3 PP4	Likely pathogenic	This study
OP10	Chr11:67811761insG	*TCIRG1*:NM_006019.4:c.971dup:p.Cys324Trpfs*166	Frameshift insertion	–	–	PVS1 PM2 PS1 PP1 PP3 PP4	Pathogenic	Sobacchi et al. 11

*MAF* minor allele frequency, *SAS* South Asian population, – absent

^**†**^v2.1.1 accessed May 7th, 2021

^**§**^Determined using wInterVar online tool (PMID: 28132688) and following the ACMG guidelines for variant interpretation (PMID: 25741868)

^¶^MAF of a different nucleotide change (G > C) at the same position is 0.000004

^¶¶^A different nucleotide change (G > C) at the same position reported as heterozygous disease variant by Allgrove, J., and Shaw, N.J. (2015)

^¶¶¶^MAF of a different nucleotide change (A > C) at the same position is 0.00001

^¶¶¶¶^Two ClinVar entries (RCV001040779, RCV000666560) exist for different nucleotide change (A > C) at the same position

PM2, Variant absent from controls or present at extremely low frequency if recessive

PP3, Multiple lines of computational evidence support a deleterious effect

PP4, Patient's phenotype or family history is highly specific for a disease with a single genetic etiology

PVS1, Null variant in a gene where loss of function is a known mechanism of disease

PM4, Variant causing protein length changes

PP1, Cosegregation with disease in affected family members in a gene definitively known to cause the disease

PS1, Same amino acid change as a previously established pathogenic variant regardless of nucleotide change

PP5, Reported as pathogenic in reputable source, but not independent evaluated

## Discussion

Here, we describe ten unrelated families with 13 affected individuals who exhibit clinical features of infantile malignant osteopetrosis and harbor likely pathogenic recessive variants in genes previously associated with osteopetrosis. In seven out of ten families (70%), we identified *TCIRG1* variants as the likely causal gene for ARO, consistent with the findings implicating *TCIRG1* as the pathogenic gene in more than 50% of ARO cases [10, 11]. The loss of function variants identified in families OP2, OP4, OP8, OP10 and splice site variants in families OP5, OP6, OP7 are predicted to result in frameshift and premature stop codons. We were not able to study the functional consequences of these variants due to unavailability of patient derived mRNA and cells. We expect the resulting products to be targeted for degradation through nonsense mediated decay or, less likely, to lead to the generation of truncated proteins that lack essential functional domains. The protein disruptive consequences of these variants implicate a loss of function as the plausible disease causal mechanism. Characteristically, these rare disruptive variants received an increase likelihood of pathogenicity in our cases (Table 2). A missense variant (TCIRG1:p.Gly172Asp) of uncertain significance identified in family OP1 affects a highly conserved amino acid residue (Additional file 1: Fig. 1) and is located in functionally crucial V ATPase I domain of TCIRG1. This variant is absent in public databases (gnomAD, 1000 Genomes Project) and predicted to be functionally damaging (SIFT; damaging, PolyPhen-2; probably damaging). However, one should be cautious in interpreting this variant until confirmed in additional ARO cases. A nonsynonymous variant in CLCN7 (p.*806Argext*58) identified in families OP3 and OP9 abolishes protein termination and expected to result in an elongated protein. We do not know the fate of this elongated protein however, CLCN7 functions as a membrane bound ion exchanger in a homodimer form [12, 13] and each abnormally elongated monomer may not adhere to conformational rearrangements required to form a membrane bound functional dimer for regulation of ion exchange. Additionally, CLCN7 forms a molecular complex with OSTM1 [14] and an elongated CLCN7 may lose the ability to form a functional complex. Further assays will be required to precisely delineate the functional consequences of CLCN7:p.*806Argext*58 variant.

According to The Human Gene Mutation Database, more than 150 ARO causing mutations are reported in TCIRG1, distributed along the entire gene and comprising all mutation types such as, missense mutations, synonymous mutations, nonsense mutations, splice defects, deep intronic variants, small insertions and deletions and large deletions [8]. Given such a high mutational heterogeneity of *TCIRG1* and absence of detailed phenotype description in some cohorts, it is difficult to establish a precise genotype–phenotype correlation in *TCIRG1* deficient ARO. Notably, *TCIRG1* mutations cause overall severe form of osteopetrosis indicated by severe hematological manifestations requiring HCST treatment. Mild to severe form of growth retardation and visual impairment are also frequently reported clinical features in *TCIRG1* deficient ARO cohorts [2]. Consistent with the reported data, the patients carrying *TCIRG1* mutations in our cohort manifest severe hematological complications and mild to severe visual impairment or normal vision at the time of diagnosis (Table 1). Similar clinical features of macrocephaly, hepatosplenomegaly and vision loss are reported in *TCIRG1* deficient ARO cases from Pakistan [15].

Two families (OP3 and OP9) in our cohort are found to carry same stoploss variant in *CLCN7* (c.2416 T > A:p.*806Arg). Both families originate from two different geographic regions although belonging to same ethnic background (Punjabi). Both families are unknown to each other and are likely unrelated. The recurrence of stoploss variant in both families could be due to either a mutational hotspot or a founder effect. The latter possibility is supported by the observation that both families originate from same ethnic background. However, with only two affected families, we have limited data to establish the possibility of founder effect for this particular locus. Genetic investigations in additional ARO cases will be required to test this possibility. Previously, two homozygous missense mutations of *CLCN7* segregating in cis in a Pakistani family with ARO was reported to exhibit dysmorphic facies, mild anemia, brain atrophy and bilateral optic atrophy [16]. Family OP3 has similar clinical features of delayed growth milestones, macrocephaly, visual impairment and mild anemia with no transfusion history. No clinical data is available for family OP9 since the tested DNA was obtained from CVS (Table 1).

Mutations in *OSTM1* are relatively less frequently (~ 5%) reported in ARO cases [8]. *OSTM1* mutations cause an osteoclast-rich, severe form of osteopetrosis with mild to severe hematological, ocular and growth defects [2]. In our cohort, only one family (OP8) carries a likely pathogenic variant in *OSTM1* and presents with clinical features of severe hematological complications, mild hepatosplenomegaly and normal vision and hearing (Table 1).

In total, we report six novel sequence variants in autosomal recessive osteopetrosis; four in *TCIRG1* and one each in *CLCN7* and *OSTM1*. Our findings suggest that *TCIRG1* is a major candidate for genetic screening of osteopetrosis in Pakistani population. Additionally, the variants identified in this study expand the mutational spectrum of autosomal recessive osteopetrosis, and will be of importance for molecular diagnosis and genetic counselling, particularly in populations with a high prevalence of autosomal recessive genetic disorders.

### Web resources

1000 Genomes Project, https://www.internationalgenome.org.

Genome Aggregation Database (gnomAD), http://gnomad.broadinstitute.org.

The Human Gene Mutation Database (HGMD), http://www.hgmd.cf.ac.uk.

## Supplementary Information

**Additional file 1.** Quality of exome sequencing analysis and conservation analysis of TCIRG1:p.Gly172.

## Data Availability

The datasets used as a reference in whole exome sequencing are available in the human genome reference assembly (UCSC Genome Browser hg19 https://genome.ucsc.edu/). Reference sequences of gene transcripts described in current study NM_006019.4 (TCIRG1), NM_014028.4 (OSTM1) and NM_001287.6 (CLCN7) can be obtained from http://www.ensembl.org/. Details of the variants analyzed during the current study are deposited in Leiden Open Variation Database (https://databases.lovd.nl/) with screening IDs 377545 (TCIRG1:NM_006019.4:c.515G > A:p.Gly172Asp), 377546 (TCIRG1:NM_006019.4:c.854_855del:p.Val285Alafs*204), 377547 (CLCN7:NM_001287.6:c.2416T > A:p.*806Argext*58), 377548 (TCIRG1:NM_006019.4:c.971dup:p.Cys324Trpfs*166), 377549 (TCIRG1:NM_006019.4:c.1165 + 1G > A), 377550 (TCIRG1:NM_006019.4:c.1554 + 2T > A), 377551 (TCIRG1:NM_006019.4:c.1555-2A > G) and 377529 (OSTM1:NM_014028.4:c.124del:p.Val42Serfs*57). The raw datasets of family members generated during the current study are not publicly available due to constraints based on informed consent, ethical concerns and institutional policy. Access to the raw sequencing data can be requested through the corresponding authors; Feng Zhang or Tahir Naeem Khan.
