# Supplementary material for: Genetic analysis of osteopetrosis in Pakistani families identifies novel and known sequence variants
Source: BMC Med Genomics. 2021 Nov 9;14:264. doi: 10.1186/s12920-021-01117-4 (PMC8576874; doi:10.1186/s12920-021-01117-4)
Supplement: Supplementary file 1 — Additional file 1. Quality of exome sequencing analysis and conservation analysis of TCIRG1:p.Gly172. [file 12920_2021_1117_MOESM1_ESM.docx]

**Supplementary data**


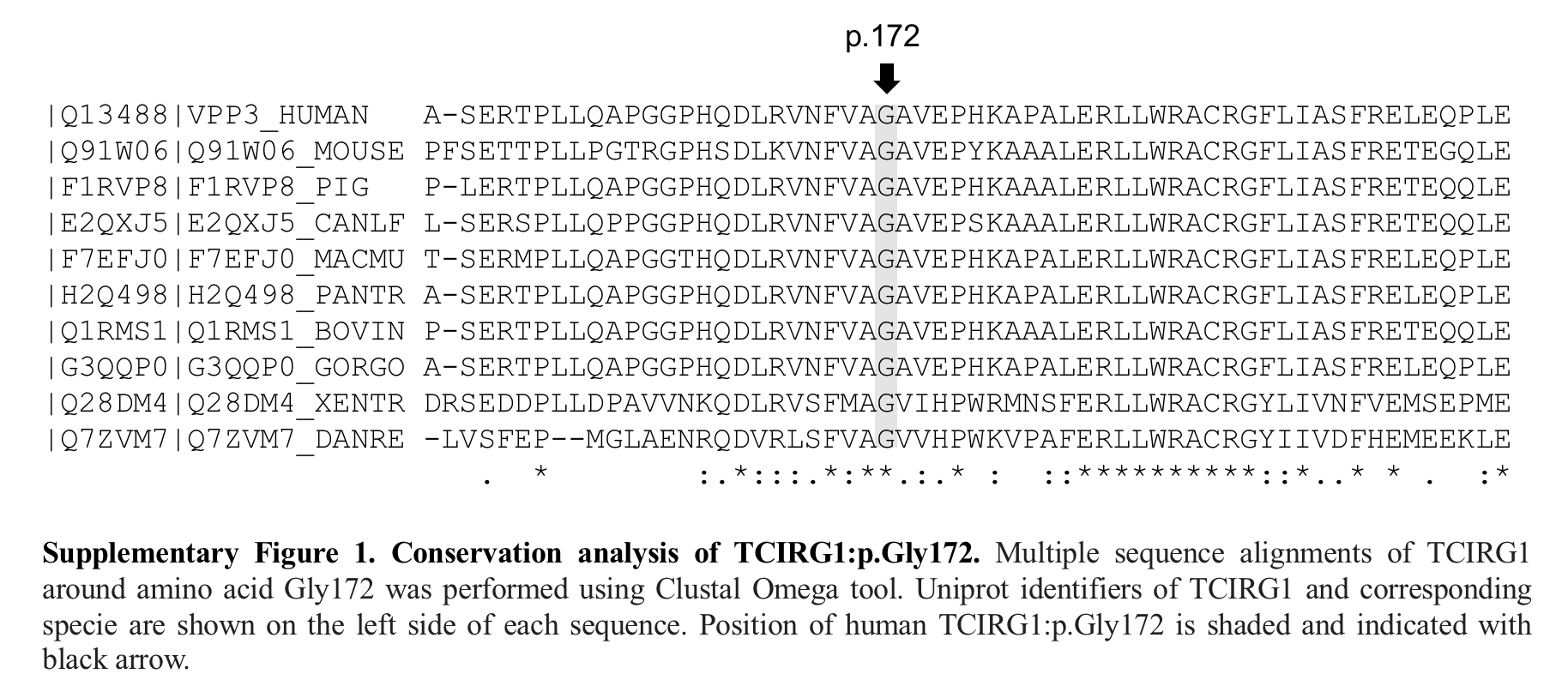


**Table S1. The quality of WES analysis for patients in this study**

| **Sample ID** | **QC rate (%)** | **Target covered mean depth** | **Coverage rate (%)** | **20X coverage rate (%)** | **30X coverage rate (%)** |
| --- | --- | --- | --- | --- | --- |
| OP1-2 | 92.51 | 131.68 | 99.87 | 96.89 | 94.14 |
| OP2-5 | 96.06 | 124.61 | 99.89 | 96.93 | 94.02 |
| OP3-3 | 95.30 | 125.50 | 99.66 | 96.82 | 93.91 |
| OP4-3 | 95.46 | 142.52 | 99.78 | 93.76 | 89.58 |
| OP4-4 | 93.44 | 145.62 | 99.86 | 94.66 | 90.67 |
| OP6-5 | 93.28 | 149.54 | 99.87 | 97.41 | 95.21 |
| OP7-3 | 94.58 | 152.46 | 99.67 | 97.58 | 95.65 |
| OP8-3 | 95.74 | 132.60 | 99.89 | 96.84 | 94.11 |
| OP10-3 | 94.97 | 155.93 | 99.79 | 95.15 | 91.74 |
